# 1,2-Bis(4-nitro­benz­yl)diselane

**DOI:** 10.1107/S1600536811025736

**Published:** 2011-07-06

**Authors:** Hua Zhou, Shi-Yi Ou, Ri-An Yan, Jian-Zhong Wu

**Affiliations:** a Department of Food Science and Engineering, Jinan University, Guangzhou 510632, People’s Republic of China

## Abstract

The title compound, C_14_H_12_N_2_O_4_Se_2_, is not chiral, but the mol­ecules assume a chiral conformation in the solid state and crystallize as an aggregate. The central C—Se—Se—C torsion angle is 90.4 (2)°, while the two Se—Se—C—C fragments assume *gauche* conformations with values of −59.4 (5) and 67.5 (4)°. The dihedral angle between the two benzene rings is 80.74 (14)°.

## Related literature

For potential applications of organoselenium compounds, see: Jung & Seo (2010 ▶). For the preparation, see: Saravanan *et al.* (2003 ▶). For related structures, see: Fuller *et al.* (2010 ▶); Lari *et al.* (2009 ▶); Hua *et al.* (2010 ▶).
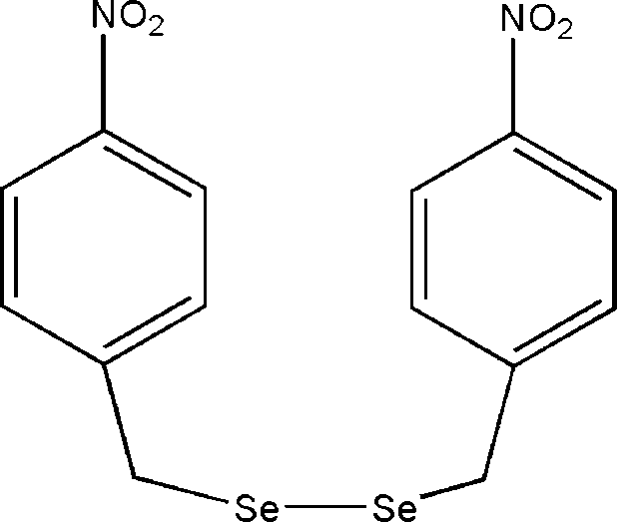


## Experimental

### 

#### Crystal data

C_14_H_12_N_2_O_4_Se_2_
*M* *_r_* = 430.18
Orthorhombic, 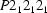

*a* = 5.88324 (14) Å
*b* = 14.3571 (3) Å
*c* = 18.3012 (4) Å
*V* = 1545.83 (6) Å^3^
*Z* = 4
Cu *K*α radiation
μ = 6.17 mm^−1^
*T* = 296 K
0.3 × 0.09 × 0.09 mm

#### Data collection

Agilent Xcalibur Gemini Ultra diffractometer
Absorption correction: multi-scan (*CrysAlis PRO*; Agilent, 2010 ▶) *T*_min_ = 0.546, *T*_max_ = 1.000
3179 measured reflections
2098 independent reflections
2015 reflections with *I* > 2σ(*I*)
*R*_int_ = 0.017

#### Refinement

*R*[*F* ^2^ > 2σ(*F* ^2^)] = 0.031
*wR*(*F* ^2^) = 0.101
*S* = 1.02
2098 reflections
199 parameters
H-atom parameters constrained
Δρ_max_ = 0.50 e Å^−3^
Δρ_min_ = −0.50 e Å^−3^
Absolute structure: Flack (1983 ▶), 659 Friedel pairs
Flack parameter: −0.02 (4)

### 

Data collection: *CrysAlis PRO* (Agilent, 2010 ▶); cell refinement: *CrysAlis PRO*; data reduction: *CrysAlis PRO*; program(s) used to solve structure: *SHELXS97* (Sheldrick, 2008 ▶); program(s) used to refine structure: *SHELXL97* (Sheldrick, 2008 ▶); molecular graphics: *SHELXTL* (Sheldrick, 2008 ▶); software used to prepare material for publication: *publCIF* (Westrip, 2010 ▶).

## Supplementary Material

Crystal structure: contains datablock(s) I, global. DOI: 10.1107/S1600536811025736/fy2016sup1.cif

Structure factors: contains datablock(s) I. DOI: 10.1107/S1600536811025736/fy2016Isup2.hkl

Supplementary material file. DOI: 10.1107/S1600536811025736/fy2016Isup3.cml

Additional supplementary materials:  crystallographic information; 3D view; checkCIF report

## Figures and Tables

**Fig. 1.**
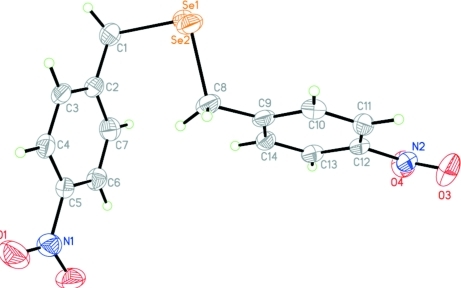
Molecular structure of the title compound. Displacement ellipsoids are drawn at the 30% probability level.

## supplementary crystallographic information

### Comment

Selenium is an important nutritional trace element involved in different physiological functions with antioxidative, antitumoral and chemopreventive properties (Jung *et al.*, 2010). Synthetic organoselenium compounds are less toxic and more chemopreventive than inorganic selenium compounds and natural organoseleniums. This is the reason why they have attracted our interest. The title compound assumes a chiral conformation in the solid state (Figure 1). The dihedral angle between the two benzene rings of the molecule is 80.74 (14)°. The C8—Se2—Se1—C1 torsion angle is 90.4 (2)°, while the Se2—Se1—C1—C2 and Se1—Se2—C8—C9 torsion angles are -59.4 (5) and 67.5 (4), respectively. All bond lengths and angles are similar to those in related structures (Fuller *et al.*, 2010; Hua *et al.*, 2010; Lari *et al.*, 2009).

### Experimental

To a vigorously stirred mixture of selenium powder (2.00 g, 25 mmol) and water (50 ml), sodium borohydride (0.95 g, 25 mmol) was added at 0 °C. The mixture was warmed to room temperature and stirred for 2 h. 1-(bromomethyl)-4-nitrobenzene (5.35 g, 25 mmol) was added and stirred for 2 h. O_2_ was passed through the solution slowly for 2 h (Saravanan *et al.* 2003). The mixture was extracted with ethyl acetate (200 ml) and washed three times with water (50 ml × 3). The obtained organic layer was dried over MgSO_4_ overnight. The organic residue was further purified by silica gel column using dichloromethane as eluent. The solvent was then evaporated and the solid residue was recrystallized from CH_3_OH to give the product as yellow crystals (yield: 4.83 g, 90%).

### Refinement

Carbon-bound H atoms were positioned geometrically and treated as riding on their C atoms, with C—H distances of 0.93 Å (aromatic) and 0.97 Å (CH_2_) and were refined with *U*_iso_(H)=1.2U_eq_(C).

### Crystal data

**Table:**

C_14_H_12_N_2_O_4_Se_2_	*D*_x_ = 1.848 Mg m^−^^3^
*M**_r_* = 430.18	Cu *K*α radiation, λ = 1.5418 Å
Orthorhombic, *P*2_1_2_1_2_1_	Cell parameters from 2222 reflections
*a* = 5.88324 (14) Å	θ = 3.1–62.7°
*b* = 14.3571 (3) Å	µ = 6.17 mm^−^^1^
*c* = 18.3012 (4) Å	*T* = 296 K
*V* = 1545.83 (6) Å^3^	Prism, metallic yellow
*Z* = 4	0.3 × 0.09 × 0.09 mm
*F*(000) = 840	

### Data collection

**Table:**

Agilent Xcalibur Gemini Ultra diffractometer	2098 independent reflections
Radiation source: Enhance Ultra (Cu)	2015 reflections with *I* > 2σ(*I*)
mirror	*R*_int_ = 0.017
Detector resolution: 16.0288 pixels mm^-1^	θ_max_ = 62.8°, θ_min_ = 5.7°
ω scans	*h* = −6→6
Absorption correction: multi-scan (*CrysAlis PRO*; Agilent, 2010)	*k* = −14→16
*T*_min_ = 0.546, *T*_max_ = 1.000	*l* = −20→20
3179 measured reflections	

### Refinement

**Table:**

Refinement on *F*^2^	Secondary atom site location: difference Fourier map
Least-squares matrix: full	Hydrogen site location: inferred from neighbouring sites
*R*[*F*^2^ > 2σ(*F*^2^)] = 0.031	H-atom parameters constrained
*wR*(*F*^2^) = 0.101	*w* = 1/[σ^2^(*F*_o_^2^) + (0.080*P*)^2^] where *P* = (*F*_o_^2^ + 2*F*_c_^2^)/3
*S* = 1.02	(Δ/σ)_max_ = 0.002
2098 reflections	Δρ_max_ = 0.50 e Å^−^^3^
199 parameters	Δρ_min_ = −0.50 e Å^−^^3^
0 restraints	Absolute structure: Flack (1983), 659 Friedel pairs
Primary atom site location: structure-invariant direct methods	Flack parameter: −0.02 (4)

### Special details

**Table:**

Geometry. All e.s.d.'s (except the e.s.d. in the dihedral angle between two l.s. planes) are estimated using the full covariance matrix. The cell e.s.d.'s are taken into account individually in the estimation of e.s.d.'s in distances, angles and torsion angles; correlations between e.s.d.'s in cell parameters are only used when they are defined by crystal symmetry. An approximate (isotropic) treatment of cell e.s.d.'s is used for estimating e.s.d.'s involving l.s. planes.
Refinement. Refinement of *F*^2^ against ALL reflections. The weighted *R*-factor *wR* and goodness of fit *S* are based on *F*^2^, conventional *R*-factors *R* are based on *F*, with *F* set to zero for negative *F*^2^. The threshold expression of *F*^2^ > σ(*F*^2^) is used only for calculating *R*-factors(gt) *etc*. and is not relevant to the choice of reflections for refinement. *R*-factors based on *F*^2^ are statistically about twice as large as those based on *F*, and *R*- factors based on ALL data will be even larger.

### Fractional atomic coordinates and isotropic or equivalent isotropic displacement parameters (Å^2^)

**Table:**

	*x*	*y*	*z*	*U*_iso_*/*U*_eq_	
Se2	3.00649 (10)	1.38512 (3)	1.48718 (4)	0.0630 (2)	
Se1	2.62212 (11)	1.35765 (4)	1.47622 (3)	0.0608 (2)	
N2	2.6203 (9)	1.7770 (3)	1.6536 (2)	0.0574 (11)	
C5	2.8508 (10)	1.5182 (3)	1.2268 (2)	0.0470 (11)	
C4	2.9801 (10)	1.4419 (3)	1.2412 (3)	0.0515 (12)	
H4	3.1209	1.4345	1.2188	0.062*	
C2	2.6858 (9)	1.3852 (3)	1.3212 (3)	0.0495 (11)	
C6	2.6351 (10)	1.5309 (4)	1.2571 (3)	0.0544 (12)	
H6	2.5479	1.5830	1.2459	0.065*	
O3	2.7429 (9)	1.8188 (3)	1.6950 (3)	0.0934 (16)	
C1	2.6009 (13)	1.3139 (4)	1.3742 (3)	0.0686 (16)	
H1A	2.6891	1.2573	1.3688	0.082*	
H1B	2.4437	1.2994	1.3630	0.082*	
O2	2.8277 (10)	1.6613 (3)	1.1697 (3)	0.0825 (13)	
C3	2.9010 (9)	1.3758 (3)	1.2891 (3)	0.0510 (11)	
H3	2.9908	1.3245	1.3004	0.061*	
N1	2.9363 (10)	1.5909 (4)	1.1763 (3)	0.0660 (13)	
O1	3.1181 (10)	1.5750 (4)	1.1456 (3)	0.0941 (16)	
C7	2.5553 (10)	1.4625 (4)	1.3047 (3)	0.0586 (13)	
H7	2.4122	1.4689	1.3257	0.070*	
C14	2.7061 (8)	1.6189 (3)	1.4953 (3)	0.0453 (10)	
H14	2.6300	1.6012	1.4530	0.054*	
C13	2.6056 (8)	1.6823 (3)	1.5430 (3)	0.0486 (11)	
H13	2.4625	1.7067	1.5330	0.058*	
C8	3.0283 (10)	1.5164 (3)	1.4576 (3)	0.0554 (12)	
H8A	3.1874	1.5330	1.4527	0.066*	
H8B	2.9576	1.5238	1.4100	0.066*	
C9	2.9178 (8)	1.5818 (3)	1.5104 (3)	0.0465 (10)	
O4	2.4153 (7)	1.7875 (3)	1.6518 (3)	0.0748 (11)	
C11	2.9349 (8)	1.6723 (4)	1.6212 (3)	0.0501 (12)	
H11	3.0131	1.6911	1.6628	0.060*	
C10	3.0275 (8)	1.6080 (3)	1.5737 (3)	0.0500 (11)	
H10	3.1677	1.5816	1.5847	0.060*	
C12	2.7213 (8)	1.7083 (3)	1.6049 (3)	0.0444 (10)	

### Atomic displacement parameters (Å^2^)

**Table:**

	*U*^11^	*U*^22^	*U*^33^	*U*^12^	*U*^13^	*U*^23^
Se2	0.0811 (4)	0.0354 (3)	0.0723 (4)	0.0084 (3)	−0.0118 (3)	0.0036 (2)
Se1	0.0845 (4)	0.0429 (3)	0.0549 (3)	−0.0137 (3)	0.0117 (3)	0.0030 (2)
N2	0.078 (3)	0.039 (2)	0.055 (2)	−0.005 (2)	0.005 (2)	−0.001 (2)
C5	0.059 (3)	0.042 (2)	0.039 (2)	0.002 (2)	0.000 (2)	−0.004 (2)
C4	0.055 (3)	0.053 (3)	0.047 (2)	0.012 (3)	0.001 (2)	−0.010 (2)
C2	0.059 (3)	0.039 (2)	0.050 (2)	−0.011 (2)	−0.005 (2)	−0.008 (2)
C6	0.057 (3)	0.052 (3)	0.054 (3)	0.013 (3)	−0.007 (2)	−0.005 (2)
O3	0.096 (3)	0.078 (3)	0.107 (4)	−0.019 (3)	0.000 (3)	−0.041 (3)
C1	0.098 (4)	0.048 (3)	0.060 (3)	−0.023 (3)	−0.007 (3)	−0.011 (2)
O2	0.109 (3)	0.054 (2)	0.085 (3)	0.006 (3)	−0.011 (3)	0.021 (2)
C3	0.065 (3)	0.037 (2)	0.051 (2)	0.008 (2)	−0.004 (2)	−0.005 (2)
N1	0.084 (3)	0.058 (3)	0.055 (2)	−0.009 (3)	−0.009 (3)	0.003 (2)
O1	0.094 (3)	0.102 (4)	0.086 (3)	−0.005 (3)	0.032 (3)	0.022 (3)
C7	0.055 (3)	0.064 (3)	0.056 (3)	−0.003 (3)	−0.003 (2)	−0.005 (3)
C14	0.050 (2)	0.038 (2)	0.048 (2)	−0.003 (2)	−0.0052 (19)	0.000 (2)
C13	0.050 (2)	0.037 (2)	0.059 (3)	−0.003 (2)	−0.003 (2)	0.006 (2)
C8	0.063 (3)	0.035 (2)	0.068 (3)	−0.004 (2)	0.007 (3)	0.001 (2)
C9	0.056 (2)	0.0276 (19)	0.055 (2)	−0.006 (2)	0.005 (2)	0.0058 (19)
O4	0.067 (3)	0.077 (3)	0.080 (3)	0.017 (2)	0.006 (2)	−0.010 (2)
C11	0.056 (3)	0.049 (3)	0.046 (2)	−0.010 (2)	−0.008 (2)	0.000 (2)
C10	0.049 (2)	0.046 (2)	0.055 (3)	0.001 (2)	0.000 (2)	0.011 (2)
C12	0.055 (3)	0.031 (2)	0.047 (2)	−0.006 (2)	0.003 (2)	0.005 (2)

### Geometric parameters (Å, °)

**Table:**

Se2—Se1	2.3043 (8)	O2—N1	1.201 (7)
Se2—C8	1.965 (5)	C3—H3	0.9300
Se1—C1	1.973 (5)	N1—O1	1.230 (8)
N2—O3	1.206 (6)	C7—H7	0.9300
N2—O4	1.216 (7)	C14—H14	0.9300
N2—C12	1.455 (6)	C14—C13	1.393 (7)
C5—C4	1.360 (7)	C14—C9	1.382 (7)
C5—C6	1.396 (9)	C13—H13	0.9300
C5—N1	1.482 (7)	C13—C12	1.374 (7)
C4—H4	0.9300	C8—H8A	0.9700
C4—C3	1.373 (7)	C8—H8B	0.9700
C2—C1	1.496 (8)	C8—C9	1.496 (7)
C2—C3	1.402 (8)	C9—C10	1.379 (7)
C2—C7	1.382 (7)	C11—H11	0.9300
C6—H6	0.9300	C11—C10	1.380 (7)
C6—C7	1.395 (8)	C11—C12	1.391 (7)
C1—H1A	0.9700	C10—H10	0.9300
C1—H1B	0.9700		
			
C8—Se2—Se1	101.78 (18)	O1—N1—C5	116.7 (5)
C1—Se1—Se2	101.5 (2)	C2—C7—C6	120.9 (5)
O3—N2—O4	123.2 (6)	C2—C7—H7	119.5
O3—N2—C12	118.5 (5)	C6—C7—H7	119.5
O4—N2—C12	118.2 (5)	C13—C14—H14	119.7
C4—C5—C6	122.4 (5)	C9—C14—H14	119.7
C4—C5—N1	119.9 (5)	C9—C14—C13	120.6 (4)
C6—C5—N1	117.7 (5)	C14—C13—H13	120.5
C5—C4—H4	120.3	C12—C13—C14	118.9 (4)
C5—C4—C3	119.4 (5)	C12—C13—H13	120.5
C3—C4—H4	120.3	Se2—C8—H8A	108.9
C3—C2—C1	120.5 (5)	Se2—C8—H8B	108.9
C7—C2—C1	120.3 (5)	H8A—C8—H8B	107.7
C7—C2—C3	119.2 (5)	C9—C8—Se2	113.3 (3)
C5—C6—H6	121.2	C9—C8—H8A	108.9
C7—C6—C5	117.6 (5)	C9—C8—H8B	108.9
C7—C6—H6	121.2	C14—C9—C8	120.3 (4)
Se1—C1—H1A	109.2	C10—C9—C14	119.0 (4)
Se1—C1—H1B	109.2	C10—C9—C8	120.7 (5)
C2—C1—Se1	112.0 (3)	C10—C11—H11	121.0
C2—C1—H1A	109.2	C10—C11—C12	118.1 (4)
C2—C1—H1B	109.2	C12—C11—H11	121.0
H1A—C1—H1B	107.9	C9—C10—C11	121.8 (5)
C4—C3—C2	120.5 (5)	C9—C10—H10	119.1
C4—C3—H3	119.8	C11—C10—H10	119.1
C2—C3—H3	119.8	C13—C12—N2	119.2 (4)
O2—N1—C5	118.3 (5)	C13—C12—C11	121.5 (4)
O2—N1—O1	124.9 (6)	C11—C12—N2	119.3 (4)
			
Se2—Se1—C1—C2	−59.4 (5)	N1—C5—C4—C3	178.7 (4)
Se2—C8—C9—C14	−102.1 (4)	N1—C5—C6—C7	−179.8 (5)
Se2—C8—C9—C10	79.7 (5)	C7—C2—C1—Se1	−75.3 (6)
Se1—Se2—C8—C9	67.5 (4)	C7—C2—C3—C4	−0.8 (7)
C5—C4—C3—C2	2.1 (7)	C14—C13—C12—N2	177.9 (4)
C5—C6—C7—C2	0.1 (8)	C14—C13—C12—C11	−0.4 (7)
C4—C5—C6—C7	1.2 (8)	C14—C9—C10—C11	−2.1 (7)
C4—C5—N1—O2	−173.5 (5)	C13—C14—C9—C8	−177.7 (4)
C4—C5—N1—O1	4.8 (7)	C13—C14—C9—C10	0.5 (6)
C6—C5—C4—C3	−2.3 (8)	C8—Se2—Se1—C1	90.4 (2)
C6—C5—N1—O2	7.5 (7)	C8—C9—C10—C11	176.2 (4)
C6—C5—N1—O1	−174.2 (5)	C9—C14—C13—C12	0.6 (7)
O3—N2—C12—C13	−159.1 (5)	O4—N2—C12—C13	22.8 (7)
O3—N2—C12—C11	19.2 (7)	O4—N2—C12—C11	−158.9 (5)
C1—C2—C3—C4	−179.2 (5)	C10—C11—C12—N2	−179.4 (4)
C1—C2—C7—C6	178.1 (5)	C10—C11—C12—C13	−1.1 (7)
C3—C2—C1—Se1	103.0 (5)	C12—C11—C10—C9	2.3 (7)
C3—C2—C7—C6	−0.2 (8)		
